# Appraising the infection prevention and control practices at two referral hospitals in Malawi: a mixed methods situational analysis

**DOI:** 10.1186/s13756-026-01742-7

**Published:** 2026-04-06

**Authors:** Dorica Ng’ambi, Thomasena O’Byrne, Wala Kamchedzera, Donald Kanjere Zgambo, Raymond Pongolani, Rufaro Matorera, Hendrina Saini, Wilned Zoto Hara, Emmie Jingini, Alex Maseko, Owen Musopole, Saulos Nyirenda, Kelvin Mponda, Gabriel Kambale Bunduki, Maryse Kok, Nicholas Feasey, Tara Tancred

**Affiliations:** 1 Department of Clinical Sciences, Liverpool School of Tropical Medicine, Liverpool, UK; 2 Malawi Liverpool Wellcome Research Programme, Kamuzu University of Health Sciences, Blantyre, Malawi; 3 Department of Dermatology, Queen Elizabeth Central Hospital, Blantyre, Malawi; 4 Quality Management Unit, Queen Elizabeth Central Hospital, Blantyre, Malawi; 5 Zomba Central Hospital, Zomba, Malawi; 6 Quality Management Directorate, Ministry of Health, Lilongwe, Malawi; 7 Centre d’Excellence en Maladies Infectieuses et Soins Critiques du Graben (CEMISoCG), Faculty of Medicine, Université Catholique du Graben, Butembo, Democratic Republic of the Congo; 8 The School of Medicine, University of St Andrews, St Andrews, UK

**Keywords:** Infection prevention and control, Healthcare associated infections, Hand hygiene, Aseptic technique, Healthcare workers, Patient-guardians, Malawi

## Abstract

**Introduction:**

Implementation of infection prevention and control (IPC) practices can reduce healthcare associated infections (HAIs). There is limited insight into the implementation of IPC practices in the medical and surgical departments in Malawian hospitals. The study aimed to explore the current state of IPC policy/guidelines and their implementation gaps at two referral hospitals in Malawi.

**Methods:**

We conducted a cross-sectional mixed-methods situational analysis to understand the IPC landscape in the medical and surgical departments in two tertiary hospitals from September 2023 to April 2024. These methods included: (i) document review; (ii) participant observation; (iii) semi-structured interviews with healthcare workers (HCWs); (iv) key informant interviews with hospital managers; and (v) focus group discussions with cleaning staff and patient-guardians. Quantitative data from participant observations were analysed in Excel to generate descriptive statistics, while framework analysis was used for qualitative data.

**Results:**

IPC guidelines were theoretically available but inaccessible to most HCWs. Observation revealed low compliance to all five moments of hand hygiene (0–12%) and non-touch technique (9–25%), often due to a lack of IPC supplies and poor knowledge. Adherence to environmental cleaning between procedures in theatre was 50%. Training of HCWs on IPC was inconsistent, and monitoring and feedback mechanisms were largely absent. There was no clear monitoring schedule for aseptic procedures, hand hygiene, or environmental cleaning. There was limited orientation on IPC practices for patient-guardians.

**Conclusion:**

We observed critical IPC gaps in both hospitals. Addressing these issues requires thoughtful implementation of multiple context-specific IPC strategies that are likely to be sustainable, such as IPC orientation for patient-guardians as they play a critical role in the Malawian healthcare system. Training of HCWs, regular monitoring and feedback on HAI/IPC practices, easily accessible IPC guidelines and improved IPC infrastructure and supplies will facilitate improved IPC practices.

**Supplementary Information:**

The online version contains supplementary material available at 10.1186/s13756-026-01742-7.

## Introduction

Infection prevention and control (IPC) comprises evidence-based practices to prevent and control the transmission of infections in healthcare facilities or settings where healthcare is provided [1, 2]. IPC aims to prevent avoidable infections in patients, patient guardians/caregivers, visitors, and healthcare workers (HCWs) by reducing the transmission of microorganisms through ensuring and promoting a safe environment [3]. Healthcare-associated infections (HAIs) are considered to be infections that patients develop at least 48 h post-admission, which were not present at the time of admission [4, 5]. The prevalence of HAIs has been estimated at around 15% across Africa, and in Malawi it is around 11.4% [4, 6, 7]. Examples of HAIs include catheter-associated urinary tract infections, intravenous cannula bloodstream-associated infections and surgical site infections. HAIs lead to longer hospital stays and increased morbidity and mortality, which strains already-challenged health systems and family resources [8]. HAIs are frequently associated with antimicrobial resistance (AMR) [9, 10], a global public health challenge that further complicates clinical management.

IPC is critical for reducing HAIs and improving patient outcomes. Standard precautions—including hand hygiene, use of personal protective equipment (PPE), environmental cleaning, aseptic techniques and waste management—form the foundation of effective IPC and should be implemented consistently across the healthcare facility [11].

Hand hygiene is the single most effective strategy to prevent HAIs, as 50–70% of HAIs are transmitted through the unclean hands of HCWs [12–16]. Ensuring availability of hand hygiene infrastructure and supplies—such as soap, clean water, and alcohol-based hand rubs—alongside routine staff training, is essential to facilitate hand hygiene.

Environmental cleaning is also crucial in preventing the transmission of microorganisms from the environment to the hands of HCWs, patients and their guardians [17]. Cleaning staff should be trained on how to perform environmental cleaning in the operating theatre and the wards [17, 18]. Microorganisms can be transmitted from the environment to the surgical wound, urethra or bloodstream through contaminated hands and the use of non-sterile materials during aseptic procedures. Adherence to aseptic techniques during invasive procedures such as wound care, urinary catheterization and intravenous (IV) cannulation by HCWs is necessary to reduce the risk of HAIs.

The World Health Organization (WHO) stresses the multimodal improvement strategy for implementation of IPC practices, which advocates for a combination of key elements. These include: availability of IPC guidelines, standard operating procedures (SOPs) and resources that are adapted to the local context; training of HCWs; monitoring and audit of IPC practices and giving feedback to HCWs; the use of reminders; and having IPC champions in a facility [16, 19, 20]. IPC guidelines and SOPs should be adapted to suit context-specific needs and be made accessible to all HCWs [2]. HCWs should be trained on the contextualised guidelines/SOPs/protocols to raise awareness and improve practices, and patients/patient-guardians should receive relevant health education [12, 20]. Training can be provided in different ways, for instance, in a classroom, on the job/bedside, through interactive sessions, as part of continuous professional development (CPD) sessions or as part of mentorship schemes [2, 21–23]. Monitoring and feedback around HCW practices related to hand hygiene, wound dressing, urinary catheterisation, IV cannulation and environmental cleaning is known to improve adherence and compliance towards IPC guidelines and protocols, leading to reduction in HAIs [2, 13, 14, 24]. It is important to develop a monitoring plan that stipulates when audits should be done, what practices or infrastructure should be audited and how feedback should be provided to relevant staff. Additionally, there must be processes in place to improve upon performance based on audit findings [20].

It is frequently the case in low-income countries that patient care by HCWs is supplemented by patient helpers, normally a close friend or family/community member of the patient who accompanies them during hospital admission. In Malawi, these helpers are termed “patient-guardians”. Their role is to assist the patient with personal care, meals and comfort. Due to HCW shortages, patient-guardians take on numerous tasks to support the patient that would, ideally, be performed by HCWs, including feeding, bathing, toileting and repositioning patients. They also report any changes in the patient’s condition to HCWs [25, 26]. However, in doing so, these patient-guardians can themselves acquire infections from the hospital environment or transmit microorganisms to-and-from the environment and the patients [27, 28].

Malawi is one of eight countries in Africa that have developed a national IPC policy and guidelines and is actively working to ensure implementation of these at the facility level [11, 29–31]. At national level IPC programmes are under the Quality Management Directorate, headed by the national IPC focal person while at facility level IPC programmes are managed by the IPC focal person and IPC committee who report to the quality manager. Most IPC activities in Malawi are funded by external partners with the ministry of health contributing only 1% of the drug budget. In Malawi, healthcare is free at the point of delivery, however annual healthcare was estimated at $39/person per year approximate three-quarters of which is from international donors funding specific programmes such as the Antiretroviral programme for HIV.

In the 2024 Joint External Evaluation report, Malawi was assessed as having a demonstrable capacity in the implementation of the IPC programme both at national and facility levels. However, not much is known about what implementation looks like in tertiary hospitals, where the risk of HAIs is highest due to increased patient and staff volumes and use of invasive procedures when compared to other facilities. The aim of this study was to describe the IPC implementation landscape at two high-volume referral hospitals in Southern Malawi to: a) understand the current state of IPC implementation; and b) identify IPC capacity gaps (both knowledge and practice) for improving IPC and reducing HAIs. This study forms part of a larger research project aimed at developing and evaluating multifaceted implementation strategies to enhance IPC practices and reduce HAIs.

## Methods

### Study context

We conducted a cross-sectional mixed methods situational analysis to understand the IPC landscape in the medical and surgical departments in two tertiary hospitals in Malawi, from September 2023 to April 2024. These hospitals were purposively selected due to their large size and tertiary status. Both hospitals are located in the region where complementary research was ongoing, which facilitated understanding of HAI prevalence in each. Hospital A is the largest referral, teaching and tertiary care hospital in Malawi and Hospital B is another tertiary referral hospital. There is limited data on the burden of HAIs and the implementation of IPC practices across these two study hospitals. Most health services in these hospitals are fully subsidised by the government. Throughout this study, we will refer to Hospital A and Hospital B to facilitate comparison and make recommendations.

### Participant sampling and recruitment

Participants were drawn from the general medical and surgical departments and included HCWs, hospital/departmental leaders, cleaning staff, and patient-guardians involved in direct patient care (Table 1).

**Table 1:** Data collection methods and participant type

Type of participant	Number	Data collection tool
HCWs	28	HCW participant observations
	17	Semi-structured interviews
Facility and departmental leaders	8	Key informant interviews
Patient guardians	32 (4 FGDs)	FGDs
Cleaning staff	16 (2 FGDs)	

*FGD* Focus group discussion, *HCW* Healthcare workers

We used purposive and convenience sampling to identify prospective participants across different categories based on their role in IPC in the hospital. Eligible participants were sampled purposively based on their role in the hospital and in IPC practices, either as a HCW or member of management or as a patient or patient-guardian. The aim was to identify: clinicians across different cadres (nurses, doctors, clinical officers, patient attendants and nurse auxiliaries) and with varying levels of work experience; managers with a specific supporting role in IPC; and inpatients and guardians of different sexes and different ages. Amongst eligible participants, we then used convenience sampling to select those who were willing and available during specific data collection periods. Participant numbers were guided by data saturation considerations for qualitative analysis, with data collection and analysis occurring concurrently until it was clear that no new insights were emerging from the data.

HCWs were recruited and observed while performing aseptic procedures (e.g. urinary catheterization, IV cannulation and wound dressing). Observation took place over a 3-week period, and all HCWs who consented to be observed and happened to be carrying out the procedures of interest during that period were included. Some of the observed HCWs, were later invited for interviews, purposively selecting from the initial cohort of observed HCWs a cross-section of cadres involved in patient care to allow triangulation of observed and reported behaviour. We also conducted semi-structured interviews with some members of management across both hospitals, who were purposively selected based on their supervisory roles in the hospital and departments.

We conducted six focus group discussions (FGDs): two with cleaners and four with patient-guardians. Patient-guardian participants were purposively sampled from those who had been in the ward for more than 2 days. We included both male and female patient-guardians of different ages. All cleaners from the wards were invited to participate in an FGD. Our data collection methods are described in more detail in Table 1.

### Data collection

We used five data collection methods: a desk review of available IPC guidelines, policies, and standard operating procedures; participant observation of IPC practices; individual interviews with HCWs; key informant interviews with facility leaders; and FGDs with cleaners and patient caregivers/guardians. Training on the data collection tools was provided by DN and TOB to research assistants—all of whom had prior experience collecting data in tertiary health facilities.

#### IPC document review

This was conducted to identify available guidelines, policies, SOPs, protocols, and visual aids on the prevention of HAIs. This involved systematically checking administrative offices, nursing stations and patient care areas within the medical and surgical departments for the presence of printed IPC documents, such as national or hospital level guidelines, protocols and visual job aids.

#### Participant observation of HCWs doing aseptic procedures

This was done to learn what HCWs do compared to what they say they do in the context of an essential and routine medical procedure. The observation tool, adapted from the CDC, WHO, and the Malawi Ministry of Health [2, 11, 32] IPC assessment tools was used to assess aseptic techniques during wound dressing, urinary catheterisation and IV cannulation (see Additional file 1). The tools were piloted to assess their feasibility and identify areas for improvement, which necessitated adding a comment section on the tool. Twenty-eight HCWs were observed performing aseptic procedures (wound dressing, urinary catheterisation or IV cannulation). To minimise the Hawthorne effect, observations were done over a prolonged period of 3 weeks to normalise the presence of the observers, and each HCW was observed performing aseptic procedure on five different patients. Further, only research assistants with a clinical and IPC background observed HCWs performing the three procedures, enabling them to blend naturally into the healthcare environment and recognize authentic versus performed behaviours [33].

#### Interviews

Semi-structured interviews were done to gain insight into HCWs’ perspectives on IPC practices. The interviews were conducted by research assistants using an interview guide informed by the desk review, facility, national and international IPC guidelines, expert knowledge, and findings from the participant observations of the aseptic techniques. We conducted interviews with seventeen HCWs who work at bedside. We also held key informant interviews with eight facility leaders.

Both tools (see Additional files 2 and 3) included questions on training provided to HCWs on HAIs, availability of IPC supplies, monitoring of IPC practices, and giving and receiving feedback about audits of IPC practices to-and-from HCWs in the wards.

All interviews were conducted in English and Chichewa by experienced research assistants familiar with the context and IPC in the participant’s office (for key informants) or in a private room in the hospital (for HCWs), lasting between 40 and 60 min.

#### Focus group discussions

FGDs were conducted with patient-guardians and cleaners to understand their perceptions of IPC practices in the wards. These FGDs were facilitated by research assistants using a guide. The desk review, participant observations, and IPC guidelines informed the FGD guide (see Additional files 4 and 5). The guide focused on their roles in the hospital, knowledge of HAIs and their prevention, hand hygiene and environmental hygiene. Six FGDs with eight participants each were done across the two hospitals: two with cleaning staff (hospital attendants and contracted cleaning staff); two with female patient guardians; and another two with male patient guardians. The patient guardians were separated by gender to allow free interaction between participants. The FGDs were conducted in a private room within the hospital, in Chichewa, and lasted between 45 and 60 min.

### Data analysis

The documents were reviewed to identify the strengths and gaps that exist for the prevention of HAIs. Data from participant observations were collected using RedCap (version 12) electronic data capturing tools [34] hosted at Malawi Liverpool Wellcome Programme (MLW) and exported as a Microsoft Excel (version 2512) file. We conducted a descriptive analysis on the exported data and presented the results as graphs and data summaries. Although our initial analysis plan only included descriptive statistics, to compare compliance between Hospital A and Hospital B across the WHO hand hygiene moments during aseptic procedures, we applied the Fishers’ exact test to identify whether there are any significant differences between the two hospitals. The Fisher’s exact test was used because of the small sample size and low compliance rates.

Audio files from interviews and FGDs were transcribed verbatim and translated into English. The transcripts were checked by DN against the audio recording for quality assurance. The transcripts were uploaded and organised in NVivo (version 12, https://www.lumivero.com/). Framework analysis was used to analyse qualitative data from the interviews and FGDs. This process involved familiarizing oneself with the data and coding it against pre-existing categories related to guidelines on core components of IPC. Within each category, individual sub-themes were generated by grouping together “child codes” into higher-order sub-themes. This particular analytical approach was useful in offering clear points of comparison between each facility and department [35].

### Ethics

The study was approved by the College of Medicine Research Ethics Committee (COMREC, P.02/23/3993) and the Liverpool School of Tropical Medicine Research Ethics Committee (LSTM REC 23-007). We obtained approvals from each hospital’s research coordinating committees and heads of departments. HCWs were briefed about the study during morning handovers and participant leaflets were posted on the hospital walls in high staff and patient traffic areas. Participant information sheets and informed consent forms (prepared in English for HCWs, and in Chichewa for patient-guardians and cleaning staff) were given to prospective participants. To enable prospective participants to give full consideration to their participation, the research assistant returned and obtained written informed consent from willing participants after a minimum of 24 h. HCWs were assured that the observations were not about them as individuals, but rather to get a picture of how aseptic procedures are typically done across each facility. We obtained verbal consent from the patients during the observations. For illiterate patient-guardians, they had a witness present as the participant information sheet and informed consent forms were read to confirm that the participant was given full study details and had the opportunity to ask questions. The witness then signed the consent form and the participant provided a thumbprint.

## Results

Data was collected from the different types of participants involved in IPC activities across both facilities. In total 101 participants took part in the study including HCWs, leaders, cleaning staff and patient guardians. Table 1 summaries the distribution of participants by type, number and data collection method.

The findings from this study reveal the state of IPC implementation in two hospitals in Southern Malawi. There is limited accessibility to guidelines, and implementation of IPC practices was inconsistent, with low compliance in hand hygiene and aseptic techniques. Our study also highlights the role of patient-guardians in IPC.

### Accessibility of IPC guidelines at the point of care

Document review was conducted to determine the availability, location and accessibility of IPC guidelines and related materials. The following facility-specific documents were identified and appraised against national and international guidelines for IPC and HAI prevention: “[Hospital A] Infection Prevention and Control Standard Operating Procedures, 2023” and “[Hospital B] IPC Policy, April 2021”. The review revealed that while general IPC guidelines were present on the ward, they were typically stored in the offices of the ward in-charges; this made the guidelines inaccessible to other HCWs (Table 2). There were some visual aids on hand hygiene and cough etiquette, which were pasted on the walls, though often not in strategic areas.

**Table 2:** Infection prevention and control (IPC) documents reviewed

Document	Author	Key findings
[Hospital A] Infection Prevention and Control Standard Operating Procedures, April 2023	IPC committee, endorsed by hospital management	Key areas for IPC are included; Procedure steps for wound dressing are partially highlighted, no visuals on the steps; Lacks details on prevention of SSIs; Hand hygiene steps are missing
Hospital A surgical checklist	Adopted by the surgical team from the WHO surgical checklist	Pasted on the walls in operating rooms to be read by a member of the surgical team before procedure; Not attached to patient files as recommended
Ministry of Health of Malawi IPC and WASH guidelines for Malawi, Nov 2020	Ministry of Health	Was used as reference material for developing the Hospital A-specific SOPs
Infection, Prevention and Control Standard Operating Procedures (in COVID-19 Contexts) in both hospitals	Ministry of Health	Guidelines on appropriate and rational use of PPE during COVID-19
[Hospital B] IPC POLICY APRIL 2021	IPC committee endorsed by the hospital management	Not accessible: only one copy with the facility IPC focal person; Key roles and responsibilities of the IPC committee are described in this policy; Monitoring and key indicators included; Standard precautions included; Continuous training and education for IPC is advocated for as one way of sharing knowledge
[Hospital B] Medical and surgical wards SOPs	2006	Outdated SOPs; Limited information on urinary catheterization and wound dressing; Comprehensive steps on intravenous cannulation
Hand washing posters at both hospitals	Endorsed by Ministry of Health	Pasted in most areas but not close to hand washing stations

*PPE* Personal protective equipment, *SOPs* Standard operating procedures, *SSIs* Surgical site infections, *WHO* World Health Organization, *WASH* Water sanitation and hygiene

These findings were corroborated by HCW interviews across both hospitals, which revealed guidelines were deemed typically inaccessible to intended users. Most of the bedside HCWs reported not having accessed the guidelines.

*But the guidelines are kept by somebody in the lockable cupboard, but we still lack those standards so that we can paste them on the board so that somebody can just follow rather than checking them in the book.* (Nurse 5—Hospital B)

However, a few participants mentioned that they have seen some guidelines.

*Lately, I have come across a manual for infection prevention, but I think it’s a recent edition that one, I think most people are not aware that it is there, most people haven’t read it probably.* (Doctor 1—Hospital A)

Despite not having access to the guideline almost all the participants highlighted the importance of having guidelines.

*Guidelines are important because they remind us of what to do. With time, you forget things, and they help you refresh and do the right thing.* (Nurse 2—Hospital A)

Furthermore, other participants expressed a need for clear and updated guidelines, SOPs and visual aids to support aseptic techniques. They felt such protocols would reinforce correct practices and address knowledge gaps. In contrast, participants in leadership roles in both hospitals offered a different perspective. While acknowledging the availability of some IPC guidelines within the hospital, they emphasised the core issue was not the absence of guidelines but the inconsistent implementation of existing protocols that operationalise the guidelines. The leaders attributed this implementation gap to behavioural challenges among staff and irregular availability of IPC resources, which hinders adherence to best practice.

*As far as I know, we have at least some guidelines on the ground, we have policies on the ground, but the biggest challenge is using them. Because if we… just follow the guidelines that we have now, I think we can be somewhere.* (KII 05—Hospital B)

### Implementation of IPC practices

We observed 28 HCWs conducting a total of 320 aseptic procedures. Of the 28 HCWs, 23 (82%) were nurses, 3 (11%) patient attendants (employed hospital support staff, who assist with wound dressing), 1 (3.5%) auxiliary nurse and 1 (3.5%) clinical officer. IV cannulation and urinary catheterisation were the most performed procedures (110, 34.4% and 120, 37.5% respectively) across both medical and surgical departments, while wound dressing was mainly observed in the surgical department. Out of the 90 wound dressing procedures, 20 were done by support staff (auxiliary nurses and patient attendants).

#### Hand hygiene practices

**Fig. 1:**
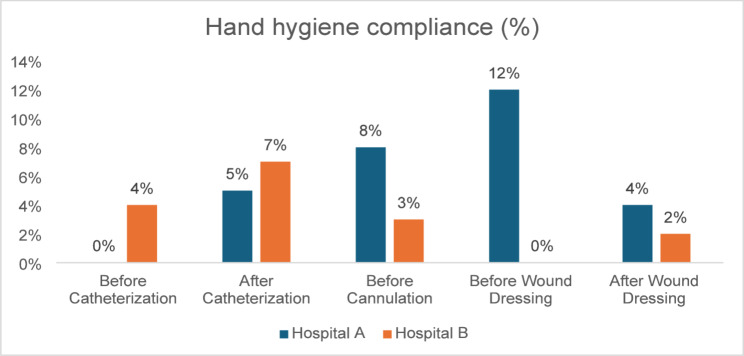
Hand hygiene compliance

Compliance was extremely low (0% to 12%, see Fig. 1) in both hospitals across all WHO moments of hand hygiene during aseptic procedures. The highest observed compliance was at Hospital A before wound dressing (12%). Fisher’s Exact Test showed no statistically significant differences between Hospital A and Hospital B at any hand hygiene moment (*p* > 0.05, see Table 3).

**Table 3:** Comparison of hand hygiene compliance (Hospital A vs Hospital B)

Hand hygiene moment	Hospital A compliant	Hospital B compliant	*p*-value*
Before catheterisation	0/28 (0%)	1/28 (4%)	1.00
After catheterisation	1/28 (5%)	2/28 (7%)	1.00
Before cannulation	2/28 (8%)	1/28 (3%)	1.00
Before wound dressing	3/28 (12%)	0/28 (0%)	0.24
After wound dressing	1/28 (4%)	1/28 (2%)	1.00

*p-value obtained from the fisher’s exact test

These findings were consistent with reports from patient-guardians across both hospitals, who noted that most HCWs do not routinely perform hand hygiene.

*Maybe if they wash their hands from their office, but when they come in the ward, I have never seen any doctor that washes his hands during the medical rounds.* (Patient-guardians FGD—Hospital A)

In line with these reports, only one HCW reported performing hand hygiene before and after procedures, indicating some individual adherence to hand hygiene practices.

*Before dressing, we wash hands, then we put on gloves, after putting on gloves, we start the wound dressing. When we are done, we take off the gloves and dispose in the bin. Then before we start another patient, we re-wash hands, after washing hands and drying them properly.* (Nurse Auxiliary—Hospital A)

During the observation period, few wards had soap, and in Hospital A there was often low water pressure. This resource limitation was highlighted by most participants as a reason for poor hand hygiene compliance.

*when we run out of resources. Like for hand hygiene, there’s no soap, there’s no sanitizer* (Nurse 5—Hospital B)

Some participants suggested that hand hygiene compliance among HCWs could be improved through peer mentoring and peer accountability. They indicated that these two strategies would encourage staff to guide and remind each other to adhere to hand hygiene practices.

#### Aseptic technique

**Fig. 2:**
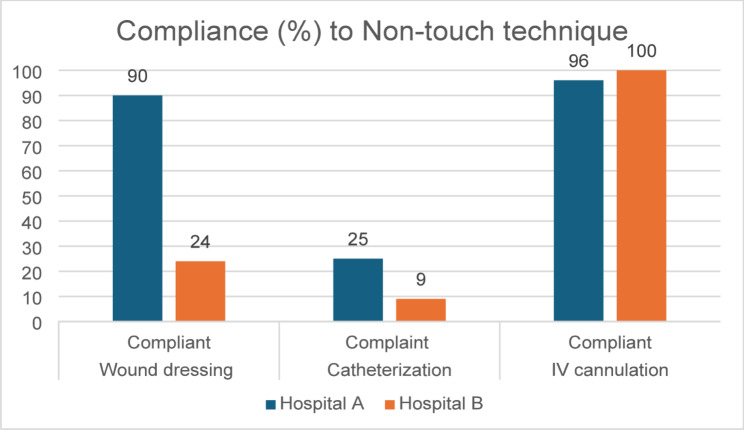
Compliance to non-touch technique

Aside from poor hand hygiene compliance, aseptic technique was variable. Hospital A had good compliance to non-touch technique during wound dressing and IV cannulation. Nurses were seen to work with and supervise the support staff doing wound dressing in Hospital A as required, which did not happen in Hospital B. Hospital B had high compliance to non-touch technique during cannulation, but very low compliance during wound dressing and catheterisation as shown in Fig. 2. The compliance rate for the use of sterile forceps was 53% (48/90) during wound dressing. However, the first step of instrument processing, which is putting the used instruments in soapy water, was done only in 8% (7/90) of the observed wound dressing procedures.

Most participants in Hospital A reported inadequate supplies, especially of sterile gloves and aprons. This led them to use examination gloves even for sterile procedures. They also reported that sometimes the use of PPE was based on the weighing the risks and benefits.

*You just weigh and act, like for example, say you have got a patient with bowel obstruction, needs to go to theatre to repair that one, and then you have no sterile gloves to insert a urinary catheter, I just go like, let me just insert the urinary catheter with the clean gloves, and then if is going to develop urinary tract infection…bowel obstruction has to go, [urinary tract infection] is treatable.* (Nurse 1—Hospital A)

During the participant observation of aseptic procedures, it was noted that documentation of these procedures in the patient files was rarely done. Only 3 out of 140 observed procedures were documented in patient files.

#### Environmental cleaning

Observations of four episodes of environmental cleaning were conducted in the main operating theatres. In 2/4 observations there was improper environmental cleaning between procedures in theatre. Preparing fresh disinfectant solution according to instructions was done in one observation. Monitors and electrical components were not always cleaned as required (compliance 2/4). A detailed operating room wash down was scheduled to be done weekly; however, over the 2 weeks of observations, it was only done in one out of the four operating rooms in the main theatre in one hospital. The other operating rooms were busy, so they could not perform the washdown.

### IPC knowledge and training of HCWs

Knowledge of IPC varied widely across both hospitals. While many health workers could mention basic IPC principles, others lacked clarity or equated IPC with routine cleaning.

*When you say IPC, we mean to say there is an element of hand hygiene, proper disposal of waste, putting on masks, putting on PPEs when you’re working and also correct use of separation of wastes as well.* (Clinician 1—Hospital B)

Training was inconsistent and often dependent on external partner support. The results from HCW interviews revealed that little or no pre- or in-service training is given to HCWs on HAIs and IPC practices. Most of the participants, especially those providing bedside care, have not been trained on IPC guidelines and HAIs.

*It’s been a long time since we got trained, I still recall something, for example, every patient should have their own dressing pack, we should avoid cross contaminating the instruments we used or patients, such interventions I can recall.* (Patient-attendant 1—Hospital B)

Most participants with leadership roles indicated the need for funds to conduct training for HCWs on IPC, HAIs and guidelines. They reported that securing funding is challenging. Sometimes they do receive funding, but it may be earmarked for a specific practice that aligns with the donor’s priorities.

*So, the key barriers are lack of knowledge, much as we say people need to be trained, I think we are not doing much, because the resources are inadequate to train everybody. You would find that the majority at this hospital are not trained.* (KII 02—Hospital A)

However, some participants suggested that IPC knowledge can be shared through other platforms such as CPD, where colleagues can engage in peer mentorship on specific topics.

*when we have new interns, we should teach them the protocols of IPC or having the CPD sessions on reminding each other about IPC.* (Clinician 1—Hospital B)

### Monitoring, audit and feedback of IPC practices

Overall, participants in leadership roles acknowledged the importance of monitoring and feedback in preventing HAIs they also reported that routine monitoring and giving feedback are often neglected due to other competing tasks and time constraints.

*Getting feedback motivates, and where you are not doing well, you know this is our challenge and we need to work on this. Sometimes a second time you see things that you were not able to see, so feedback is important because you can reflect and then sort things out.* (KII 04—Hospital A)

Most HCWs reported that monitoring of IPC practices is not conducted, and even when it is conducted, it is rarely followed by feedback. Bedside HCWs want feedback to be given in a constructive manner, not a punitive way, and to recognise positive practices alongside areas for improvement. A few participants suggested assigning an IPC focal person in each ward could enhance monitoring efforts and ensure regular supportive feedback on IPC compliance.

*So, we really need to have focal persons in IPC in each ward and a team that is looking at IPC, which is effective, which should be meeting frequently, maybe once a month to look at how we are doing with IPC.* (Nurse 1—Hospital A)

### The role of patient guardians in IPC

There is little-to-no orientation on IPC practices for patient-guardians and this affects the way guardians behave while in the wards. During FGDs, only a few patient-guardians reported to have received an orientation of the ward environment. Most of the patient-guardians indicated that they were only given a bed for the patients, without being oriented to the ward surroundings. The HCWs agreed on the need to orient patient-guardians but indicated they do not have enough time to give health talks to guardians and their patients due to staff shortages.

*The issue is the same, about shortage, you see that you have a lot of work to do and then to think of standing there shouting “guardians come over here to learn for 30 min”, you feel like those 30 min are wasted, instead you could have done something*. (Nurse 2—Hospital A)

FGDs with patient-guardians revealed the presence of a guardian chairperson who serves as a liaison between fellow guardians and HCWs. The participants agreed that this chairperson could play a key role in orienting new guardians upon arrival. Additionally, some participants suggested that the cleaners or security guards could help in providing initial orientation within the wards.

*So it cannot only be a job of the nurses only, and they can even be choosing a guardian chairperson to be following up with that, because some guardians have been in the hospital with their patients for more than a month or two months, so those people they can also be in a better position to make sure that every other guardian is being responsible when they use the bathrooms and the toilets*. (Patient—guardian FGD—Hospital B)

## Discussion

Our study describes the implementation of IPC guidelines and practices for preventing HAIs in two referral hospitals in Malawi. Overall, there is a low level of IPC programme implementation across both hospitals. Guidelines and SOPs for the prevention of HAIs among HCWs are not widely available, there is a lack of training for HCWs on IPC, and there is a lack of health education or orientation for patient-guardians on IPC on the general ward environment. Another key finding was that supervision and monitoring of IPC practices and giving feedback to staff were irregular. This was accompanied by low compliance to hand hygiene and aseptic techniques.

Guidelines/SOPs are crucial for preventing HAIs, by providing clear guidance on standard precautions and IPC practices to follow when conducting aseptic procedures. Moreover, they can serve as accountability tools by setting expectations and responsibility for HCWs’ actions. Potential ways to increase guideline/SOP availability and use could include raising awareness among HCWs on the guidelines, distributing electronic copies of the guidelines and evaluating the implementation of these guidelines to improve IPC practices and reduce HAIs, as has been carried out with success elsewhere [20, 36].

Proper hand hygiene is a critical component for the prevention of HAIs [8, 16, 37]. Improving hand hygiene practices through training of HCWs on hand hygiene, use of reminders and improving availability of hand hygiene facilities and resources have been found to encourage HCWs to perform hand hygiene in other African settings [16, 38, 39]. Consistent with our findings, barriers such as inadequate resources, lack of knowledge and poor attitudes to hand hygiene have been reported elsewhere [37]. This finding emphasises the need for adequate provision of hand hygiene resources and positive HCW attitudes in order to improve hand hygiene compliance.

Adherence to aseptic technique was inconsistent, with poor compliance with non-touch technique, particularly during wound dressing and urinary catheterisation. Active involvement of nurses in supporting and supervising support staff during wound dressing procedures was found to be a facilitator for good aseptic practices among the support staff. This collaborative approach likely reinforces correct technique and adherence to IPC standards highlighting the potential value of structured nurse–support staff mentorship as a strategy to improve and sustain IPC practices. Evidence indicates that mentorship programs reduce clinical errors, support professional development and promote quality care [40, 41].

Some HCWs reported using examination gloves instead of sterile gloves, which reflects the risks associated with irregular supply and inadequate evidence-based training. Non-compliance with aseptic techniques during invasive procedures creates opportunities for microorganisms transfer from HCWs’ hands, non-sterile gloves and environment to patients, thereby increasing the risk of HAIs [42]. A study conducted in Bangladesh similarly revealed that the high HAI prevalence was related to inadequate aseptic resources and poor IPC practices [43]. There is a need for interventions to improve adherence to aseptic techniques to improve clinical outcomes for individuals and healthcare quality more broadly related to aseptic procedures.

Our study revealed gaps in IPC training for ward-based HCWs; most HCWs had not received formal pre-service and in-service IPC training. Our findings align with the 2024 Malawi Joint External Evaluation report, which highlighted the lack of a formal pre-service IPC module in various health-related courses, except for nursing, offering training on standard precautions. This lack of training could also contribute to poor adherence to hand hygiene and non-touch technique among bedside HCWs. Knowledge improves compliance with IPC practices, which in turn leads to the prevention of HAIs. Many studies [18, 24, 38, 44] have highlighted the importance of training HCWs on IPC and the prevention of HAIs. Singh et al. [45] reported that modular in-service training reduced SSI rates from 46% to 3.27% per 100 surgeries in a cardiovascular surgery unit in India, showing the importance of providing regular training and education to HCWs in order to reduce HAIs. Our study further highlights the need for ongoing training and repeated reinforcement of key IPC messages as essential strategies for sustaining behavioural change among HCWs.

Our study revealed poor monitoring of IPC practices and that feedback was not given to staff, even when monitoring was undertaken across both hospitals. Monitoring, audits and giving feedback is a core component of IPC that is essential to improve IPC practices and reduce HAIs [2]. HCWs in low-resource settings tend to change their IPC practices once they have been given feedback about how they are performing [5, 8, 17, 18, 24]. Monitoring of IPC practices such as hand hygiene, aseptic procedures or environmental cleaning and sharing performance data is essential to improve practices and reduce HAIs. Regular monitoring should be done by facility management, IPC focal persons and IPC committees and feedback should be given to relevant staff in support of their CPD [20].

In Malawi, patient-guardians play a critical role in caring for patients—a common practice across Africa. Patient-guardians should, therefore, be involved in IPC implementation by orientating them on hand hygiene and to the ward environment, including bathroom facilities and waste management. Hospitals must ensure clean wards, functional sinks and toilets and adequate hand hygiene resources [2, 11]. In both hospitals, our study revealed that patient-guardians are not given enough information on infection transmission and how HAIs can be prevented. This is consistent with findings in other settings, for instance, a study in Ethiopia found that health education was given to clients and visitors only four times instead of the planned 48 times per year [46]. Lack of information on personal and environmental hygiene in the healthcare setting for patient-guardians can lead to failure to prevent transmission of infections from the environment to patients and to themselves. This transmission can be prevented if the patients and patient-guardians are given adequate health education on the prevention of infections, hand hygiene, waste management and environmental cleaning [8, 25]. Our study found that, in contexts where HCWs are overstretched, orientation for patients and their guardians can be delegated to trained non-clinical staff. This highlights the need to identify effective and sustainable ways of delivering orientation and health education to patient-guardians.

These findings should be considered in light of the following limitations: the study was conducted in two hospitals, which limits the generalisability of the findings to other healthcare facilities in Malawi. The presence of research assistants during observation might have introduced bias due to the Hawthorne effect. However, our findings suggest that their presence had minimal influence on the actual performance of the HCWs during their duties. Overall, mixed methods were used to facilitate the explanation of quantitative findings derived from participant observations through qualitative methods, increasing rigour and trustworthiness of findings through triangulation [47].

### Implications for practice and policy in Malawi

- The study hospitals, and more broadly, the Ministry of Health should set aside a dedicated budget for procurement and provision of necessary equipment and commodities needed for implementing IPC activities. Findings from the WHO global IPC survey [7] indicate countries with a specific IPC budget demonstrated measurable improvements in IPC structure and implementation.
- IPC guidelines and SOPs should be made easily accessible to bedside HCWs. Some studies [48, 49] have reported an association between the availability of guidelines and awareness of the guidelines among HCWs with improved adherence.
- Alternative platforms for knowledge sharing such as CPD and handover meetings should be promoted to improve HCWs’ understanding of IPC and HAIs. Zhang et al. [50], noted that CPD equips HCWs with knowledge and skills on IPC guidelines and emerging evidence, supporting adherence to recommended IPC measures.
- There should be a designated IPC focal person to lead in conducting IPC audits, monitoring and providing feedback of the audited practices to staff and hospital management. Patel et al. [51] found that giving monthly feedback to staff improved hand hygiene compliance from 34 to 76%.
- A health education package for patient-guardians should be developed/adapted, and HCWs should be trained on how this information can be delivered to them. Basu and colleagues [25] described how structured health education given to the patient-guardians can lead to improved IPC practices and that this health education was highly valued.

## Conclusion

There is currently limited implementation of IPC practices in both medical and surgical departments in our study sites in Malawi. In line with WHO recommendations to use multimodal strategies for IPC implementation, having easily accessible IPC guidelines and standard operating procedures, training HCWs, orientating patients and their guardians around IPC measures, improving IPC resource availability and supporting monitoring and feedback of IPC activities would collectively assist in improving IPC practices and reducing HAIs. There is a need to develop appropriate strategies for the Malawian context to improve implementation and sustainability of IPC practices for the prevention of HAIs, with particular recognition of the key role patient-guardians play in patient care.

## Supplementary Information

Below is the link to the electronic supplementary material.

Supplementary Material 1

Supplementary Material 2

Supplementary Material 3

Supplementary Material 4

Supplementary Material 5

## Data Availability

The datasets used and/or analysed during the current study are available from the corresponding author on reasonable request.
