# Supplementary material for: Appraising the infection prevention and control practices at two referral hospitals in Malawi: a mixed methods situational analysis
Source: Antimicrob Resist Infect Control. 2026 Apr 6;15:76. doi: 10.1186/s13756-026-01742-7 (PMC13188669; doi:10.1186/s13756-026-01742-7)
Supplement: Supplementary file 1 — Supplementary Material 1 [file 13756_2026_1742_MOESM1_ESM.pdf]

MALAWI LIVERPOOL WELLCOME PROGRAMME

KAMUZU UNIVERSITY OF HEALTH SCIENCES

**IV cannula insertion**						
**Observed criteria**	• **Verification**	**Patient 1**	**Patient 2**	**Patient 3**	**Patient 4**	**Patient 5**
Preparation	• Explains the procedure to the patient					
	• Prepares necessary equipment and supplies					
Hand hygiene before insertion	• Hand hygiene is performed correctly<br>• Hand wash<br>• Alcohol based hand rub					
Pre- insertion tasks are performed properly to prevent infection	• Checks the IV solution (bottle or plastic bag) to be sure it is correct					
	• Uses “non-touch” technique to open infusion set					
	• Inserts infusion set into solution bottle or bag without touching the opening and the spike					
	• Fills the infusion chamber and tubing					
	• Positions the arm with support					
	• Applies tourniquet above					
The provider performs antisepsis before inserting the IV line.	• Puts on clean disposable gloves					
	• Using a fresh cotton swab, cleans the site with antiseptic in a circular motion starting at the centre					
	• Allows antiseptic to dry					
	• Maintains sterility at the insertion site after antiseptic is applied					
Insertion	• Inserts needle with the bevel up into vein without touching other surfaces.					
	• Looks for blood return in the tubing and carefully advance the needle					

	• Releases the tourniquet					
	• Secures cannula with adhesive tape/ transparent dressing.					
	• Labels (date, time, initial) the IV insertion site (on the plaster), bottle or bag with the type of additives					
Healthcare waste management	• Disposes of healthcare colour coded/labelled waste in a correct container					
IPC practices are implemented to reduce the risk of infection related to IV lines.	• Labelling (date, time, initial)					
	• Condition (infiltration, swelling, redness, pus)					
	• Dressing is dry, clean, and not tight					
	• The infusion site is changed every 72–96 hours					

**Urinary catheterization**						
**Observed criteria**	**Verification**	**Patient 1**	**Patient 2**	**Patient 3**	**Patient 4**	**Patient 5**
Preparation	• Explains the procedure to the patient					
	• Prepares necessary sterile and clean equipment and supplies					
	• Positions the patient					
Hand hygiene before catheterization	• Hand hygiene is performed correctly<br>○ Hand wash<br>○ Alcohol based hand rub<br>•					
Pre- insertion tasks are performed properly to prevent infection.	• Opens catheterization package, keeping bottom of pack sterile					
	• Puts on sterile gloves					
	• Organizes supplies on sterile field					
	• Applies lubricant along sides of catheter tip					

The provider performs the antisepsis before inserting the catheter.	• Cleans urethral meatus with a non-alcoholic antiseptic solution:<br>○ If female: 5 swab technique<br>○ male: retracts and cleans the foreskin (if uncircumcised), cleans glans and urethral opening					
Insertion	• Uses "non-touch" technique					
	• Allows bladder to empty					
	• Inflates balloon					
	• Attaches end of catheter to a close collection system					
	• Secures the catheter to the thigh					
	• Ensures that there is no obstruction or kinks in tubing					
	• Ensures that the urinary bag is below the level of bladder					
	• Checks that the urinary bag is above the floor					
Healthcare waste and instrument management	• Disposes of healthcare waste in a colour coded/labelled container					
	• Puts reusable dirty instruments in soapy water					
	• Removes gloves and disposes in a leak proof container					
Hand hygiene	• Washes hands with soap and water					
	• Allows hands to dry					
	• Documents the indication, date and time					

MALAWI LIVERPOOL WELLCOME PROGRAMME

KAMUZU UNIVERSITY OF HEALTH SCIENCES

Wound management (during ward round and wound dressing						
**Observed criteria**	• **Verification**	**Patient 1**	**Patient 2**	**Patient 3**	**Patient 4**	**Patient 5**
Preparation	• Prepares necessary supplies					
	• Explains the procedure to the patient					
	• Wears appropriate PPE					
	• Protects the area surrounding the patient's wound with a plastic sheet					
Hand hygiene before wound dressing.	• Hand hygiene is performed before the procedure:<br>• Wash hand<br>• ABHR					
Removal of old wound dressing	• Puts on clean examination gloves					
	• Removes old wound dressing and disposes correctly					
	• Pours sterile normal saline if dressing is adherent					
	• Removes gloves and disposes correctly					
	• Washes hands					
New wound dressing	• Puts on sterile gloves					
	• Uses sterile forceps, cotton balls or gauze to clean the wound (non-touch technique)					
	• Applies prescribed dressing solutions or medicines					
	• Applies sterile dressing, one layer at a time					
	• Secures the dressing					
Healthcare waste and instruments management	• Disposes of healthcare waste in a colour coded/labelled correctly					
	• Places instruments in soapy water					
	• Removes gloves and disposes them correctly					
Hand hygiene after wound dressing	• Washes hands with soap and water					
Preoperatively						

No shaving of the operation site	• Checks patient file if patient was told not to shave					
	• If prophylaxis antibiotic was administered timely					
