# Supplementary material for: Appraising the infection prevention and control practices at two referral hospitals in Malawi: a mixed methods situational analysis
Source: Antimicrob Resist Infect Control. 2026 Apr 6;15:76. doi: 10.1186/s13756-026-01742-7 (PMC13188669; doi:10.1186/s13756-026-01742-7)
Supplement: Supplementary file 2 — Supplementary Material 2 [file 13756_2026_1742_MOESM2_ESM.pdf]

Appendix 2 Interview Topic Guide A: Healthcare Workers

Appendix 2 Interview Topic Guide A: Healthcare Workers

NB The structure and questions of the interviews may vary from one participant to the other depending on the job role/experience. The topic guide should ideally evolve over time. Possible topic areas therefore include the following)

SSI Topic Guide

PART 1: PARTICIPANT DETAILS

Record the details for each participant.

PART 2: INTERVIEW INTRODUCTION

**Hospital ID** [______]	**Interviewer initials** [___|_____|____]
**Interview ID number** [_____|______|______]	**Note-taker initials (if present)** [___|_____|____]
**Date:** [___|___] / [___|_____] / - [_____|_____] day month year	**Time start** [_____|_____]: [_____|______]<br>**Time end** [_____|_____]: [_____ |_____]

Introduction

I am ________________________________ from _______________________ (Interviewer)

Thank you very much for taking the time to speak to me today. My name is [} and I am one of the IPC-Implement study team members. Before we begin, can I please confirm that you have received a copy of the study information sheet and consent form?

As a reminder, this study aims to explore how we can improve infection prevention and control in Malawi (IPC) to improve patient safety and quality of care. There are no right or wrong answers. Everything you say will be treated confidentially and will not be shared with any of your colleagues, or anyone outside of the IPC-Implement study team. You are free to answer in as much or as little detail as you wish, to skip over any questions you do not wish to answer, and to pause or stop the interview at any time if needed.

This interview will take approximately one hour- depending on how much you have to say. Can I please check you are free at the moment to talk for this amount of time?

I would also like to please record our conversation- so that I can capture your responses

Approved by
College of Medicine
17-May-2023
(COMREC)
Research and Ethics Committee

accurately, and so that I can listen to you rather than take many notes. Can I confirm you are happy for me to start recording? Great. Thank you.

We would like to start with understanding a bit about your role in your current job and the facility where you work:

1. What is your current position? How long have you been in this position at this facility?
2. Could you briefly tell me a bit about how the surgical/medical ward in your facility is set-up?
3. Which parts of the surgical/medical ward do you currently work on? Do you currently work in any other ward?*: if yes, what do you do?*

Thank you for sharing. For the remainder of this interview, I would like to focus on discussing *healthcare associated infections (HAIs) and infection prevention and control (IPC) in your facility*

4. What do you understand by the term quality of care? And what do you understand by the term patient safety?
5. Could you tell me a bit about your understanding of what healthcare associated infections are?
6. How much of an issue do you think HAIs are at this hospital? Within the surgical/medical wards? What is it that makes it an issue or not an issue?
7. Could you tell me a bit about your understanding of infection prevention and control?
8. How much of an issue do you think IPC is at this hospital? Within the surgical and medical wards? What is it that makes it an issue or not an issue?
9. Overall, is there anything that makes it difficult for you and your colleagues to practice IPC? What gets in the way sometimes?
10. Can you describe an example of when IPC was challenging? What made it challenging? What would make it easier?

11. Can you provide an example of when IPC worked well? What was successful about it?

12. How much of a priority is infection prevention and control? *Why is that?* Is there anything at a higher priority? Have you received any training about the IPC? Is this in addition to that which you may have received back in [medical/nursing/midwifery] school?

    When was the training? Where was the training? (in facility or external); what did the training cover; how was it delivered? (in classroom, online), Did you find it helpful/not helpful?

13. Do you feel any additional training is needed on IPC? If so, what would you like to receive training on?

14. Do you use any clinical guidelines or protocols for IPC in your facility? If so, which ones?
    Prompt: local or national guidelines?
    If guidelines are used: Are they displayed? If not displayed, why and where are they

Approved by
College of Medicine
17-May-2023
(COMREC)
Research and Ethics Committee

physically located? Is it easy/not easy to access them? How often or when would you use these guidelines/protocols? How useful are guidelines your clinical practice? Why is that?

If guidelines are not used? Why not?

15. Do you feel more guidelines and protocols for IPC are needed? *Prompts: Why is that? What types of guidelines or protocols are needed?*
16. How is IPC monitored in this facility?
17. In your opinion, do you work well as a team when trying to implement IPC? Why or why not? *Prompts: In general, what gets in the way of team working? What would help you work better as a team when trying to improve IPC?*

10. Do you lack any resources needed to manage this IPC? *Time, staffing, supplies? Was everything easily available when you needed it? What happens if what you need is not there or is not working? Anything else about the environment working conditions e.g., bed occupancy, workload etc.*
11. How do you communicate with patients/guardians/staff about IPC?
12. Tell me about the role that you think guardians/visitors/patients play in IPC? What do you think could help improve IPC as far as the community is concerned?
13. Have you ever been concerned about complaints from the community about the care they received? How does this impact on you and your colleagues?

Thank you for your responses. We are now moving on to the final section of the interview where I have a few more general questions about IPC

14. Does your team get any feedback on how IPC on the wards? *If yes, ask* what does the feedback cover? How useful do you feel this feedback is? What could be done to make this feedback more useful?
15. In your opinion, what do you think would happen if IPC was not managed well in the wards? *Would there be any consequences? For whom?*
16. Are you aware of any strategies in your facility to try to improve IPC? If yes, please explain and in your opinion, how effective have these strategies been?
17. Finally, is there anything else that you would like to share with me about anything we discussed today?

Approved by
College of Medicine
17-May-2023
(COMREC)
Research and Ethics Committee
