# Supplementary material for: Appraising the infection prevention and control practices at two referral hospitals in Malawi: a mixed methods situational analysis
Source: Antimicrob Resist Infect Control. 2026 Apr 6;15:76. doi: 10.1186/s13756-026-01742-7 (PMC13188669; doi:10.1186/s13756-026-01742-7)
Supplement: Supplementary file 3 — Supplementary Material 3 [file 13756_2026_1742_MOESM3_ESM.pdf]

Appendix 3: Semi-Structured interview guide: IPC Stakeholders

Appendix 3: Semi-Structured interview guide: IPC Stakeholders

NB The structure and questions of the interviews may vary from one participant to the other depending on the job role/experience. The topic guide should ideally evolve over time. Possible topic areas therefore include the following)

SSI Topic Guide	
**PART 1: PARTICIPANT DETAILS**	
Record the details for each participant.	
**PART 2: INTERVIEW INTRODUCTION**	
**Organisation ID** [______]	**Interviewer initials** [___|_____|_____]
**Interview ID number** [_____|______|______]	**Note-taker initials (if present)** [___|_____|_____]
**Date:** [___|___] / [___|_____] / - [_____|_____] day month year	**Time start** [_____|_____]: [_____|______] **Time end** [_____|_____]: [_____ |_____]

Introduction

I am _________________________________ from ________________________ (Interviewer)

Thank you very much for taking the time to speak to me today. My name is [ ] and I am one of the IPC-Implement study team members. Before we begin, can I please confirm that you have received a copy of the study information sheet and consent form?

As a reminder, this study aims to explore how we can improve infection prevention and control in Malawi (IPC) to improve patient safety and quality of care. There are no right or wrong answers. Everything you say will be treated confidentially and will not be shared with any of your colleagues, or anyone outside of the IPC-Implement study team. You are free to answer in as

Approved by
College of Medicine
17-May-2023
(COMREC)
Research and Ethics Committee

Appendix 3: Semi-Structured interview guide: IPC Stakeholders

much or as little detail as you wish, to skip over any questions you do not wish to answer, and to pause or stop the interview at any time if needed.

This interview will take approximately one hour- depending on how much you have to say. Can I please check you are free at the moment to talk for this amount of time?

I would also like to please record our conversation- so that I can capture your responses accurately, and so that I can listen to you rather than take many notes. Can I confirm you are happy for me to start recording? Great. Thank you.

Background

We would like to start with understanding a bit about your job and where you work:

1. What is your current position? How long have you been in this position? What is your background?
2. Could you briefly tell me a bit about your understanding of the health system here in Malawi?

Thank you for sharing. For the remainder of this interview, I would like to focus on discussing *healthcare associated infections (HAIs) and infection prevention and control (IPC)*

3. What do you understand by the term quality of care? And what do you understand by the term patient safety?
4. Could you tell me a bit about your understanding of what healthcare associated infections are?
5. How much of an issue do you think HAIs are in Malawi? National level and facility level? What is it that makes it an issue or not an issue?
6. Could you tell me a bit about your understanding of infection prevention and control?
7. What do you know about the core components of IPC?
8. What guidelines are you aware of/do you have related to IPC/WASH/AMR/AMS?
9. What policies and standards are you aware of/do you have related to IPC?
10. Do you feel more guidelines and protocols for IPC are needed? *Why is that? What types of guidelines or protocols are needed?*
11. Can you share copies of any IPC related documents that you have?
12. What do you know about the national IPC plan in Malawi/beyond? What are your priority areas related to IPC in your organisation/work?
13. What parts of MoH take responsibility for the different components of IPC and how? How is IPC monitored in Malawi/your organisation?
14. Who are the partners working with the government on IPC activities? Do they have specific areas or districts where they work? Tell me more on what you know about these activities or new strategies for IPC. Please explain and in your opinion, how effective have these strategies been?

Approved by
College of Medicine
17-May-2023
(COMREC)
Research and Ethics Committee

Appendix 3: Semi-Structured interview guide: IPC Stakeholders

Barriers and facilitators to IPC

15. How much of an issue do you think IPC is in Malawi in health facilities? What is it that makes it an issue or not an issue?

16. What do you think are the key barriers/facilitators to IPC in Malawi? *Probe on the 8 core components if not already mentioned (IPC programme; IPC guidelines; education and training; surveillance; multimodal strategies; monitoring/audit of IPC practices and feedback; workload, staffing and bed occupancy; built environment, materials and equipment for IPC at the facility level)*
17. What makes it difficult for you and your colleagues to improve IPC? What gets in the way sometimes?
18. Can you describe an example of when IPC was challenging?
    What made it challenging? What would make it easier?

19. Can you provide an example of when IPC worked well? What was successful about it?

20. How much of a priority is infection prevention and control? *Why is that?* Is there anything at a higher priority? Have you received any training about IPC?

    If yes, or if they know if training When was the training? Where was the training? (in facility or external); what did the training cover; how was it delivered? (in classroom, online), Did you find it helpful/not helpful?

21. Do you feel any additional training is needed on IPC? If so, what would you like to see training on?

22. Tell me about the role that you think guardians/visitor/ patients play in IPC? What do you think could help improve IPC as far as the community is concerned?
23. Are you aware or have you ever been concerned about complaints from the community about the care they received? How does this impact on you/your colleagues/health system?

Conclusion

Thank you for your responses. We are now moving on to the final section of the interview where I have a few more general questions about IPC and future plans for the project

24. Do you or your team provide or get any feedback on how IPC ? *If yes, ask* what does the feedback cover? How useful do you feel this feedback is? What could be done to make this feedback more useful?
25. How do you communicate with others about IPC?
26. Do you have any suggestions on areas for research in IPC?
27. Who else do you think we should be talking to?

Approved by
College of Medicine
17-May-2023
(COMREC)
Research and Ethics Committee

Appendix 3: Semi-Structured interview guide: IPC Stakeholders

28. How do you think we could collaborate in this work*? Talk about the stakeholders meeting/workshop*. Do you have any suggestions for this and would you be interested in coming?
29. Finally, is there anything else that you would like to share with me about anything we discussed today?

Approved by
College of Medicine
17-May-2023
(COMREC)
Research and Ethics Committee
