# Supplementary material for: Appraising the infection prevention and control practices at two referral hospitals in Malawi: a mixed methods situational analysis
Source: Antimicrob Resist Infect Control. 2026 Apr 6;15:76. doi: 10.1186/s13756-026-01742-7 (PMC13188669; doi:10.1186/s13756-026-01742-7)
Supplement: Supplementary file 4 — Supplementary Material 4 [file 13756_2026_1742_MOESM4_ESM.pdf]

Zoonjezera 7a ndi 7b: Zokambirana za pa gulu: Chikalata chotsogolera zokambirana: Wogwira ntchito yosamalira pa malo

Chikalata chotsogolera zokambirana chikuyenera kusintha pakadutsa nthawi kutengera uthenga wina umene watoleredwa kuphatikizapo zimene zaonedwa. Choncho mitu yokambirana ikhoza kukhala yotsatirayi:

Chikalata cha Zokambirana za pagulu (FGD) za wogwira ntchito yosamalira pa malo	
**GAWO 1: UTHENGA WOKHUDZANA NDI WOTENGA NAWO MBALI**	
Lembani uthenga wokhudzana ndi wotenga nawo mbali wina aliyense.	
**GAWO 2: MAU OYAMBA A ZOKAMBIRANA ZA PA GULU (FGD)**	
**ID ya Chipatala** [______]	**Zilembo zoyambilira za dzina la wotsogolera zokambirana** [___|_____|_____]
**Namabala ya ID ya zokambirana za pa gulu** [_____|______|______]	**Zilembo zoyambilira za dzina la wolemba zokambidwa pa mchezo (ngati alipo)** [___|_____|_____]
**Tsiku:** [___|___] / [___|____] / - [_____|_____]<br>tsiku mwezi chaka	**Nthawi yoyambira** [_____|_____]: [_____|______]<br>**Nthawi yomalizira** [_____|_____]: [_____ |_____]

Mau oyamba

Ine ndi ________________________________ ndikuchokera ku _______________________ (Wotsogolera)

Ine ndi ________________________________ ndikuchokera ku _______________________ (Wolemba zokambirana pa mchezo)

Zikomo kwambiri chifukwa chotenga nthawi yanu kulankhula ndi ine lero. Dzina langa [ ] ndipo ndine m'modzi wa mugulu la kafukufuku wa IPC-Implement. Tisanayambe zokambiraa, ndikufuna nditsimkize ngati mwalandira tsamba la chikalata cha uthenga ndi chikalata cha chilolezo?

Mongokumbutsa, kafukufukuyu cholinga chake ndikufufuza m'mene tingapitisire patsogolo njira zopewera kutenga ndi kuchepetsa matenda m'Malawi (IPC) ndi choliga chofuna kupitisa patsogolo chitetezo ndi chisamaliro chabwino kwa odwala. Takupemphani kuti mutenge nawo mbali mu zokambirana za tsiku la

Approved by College of Medicine
17-May-2023
(COMREC)
Research and Ethics Committee

lero chifukwa tikuyembekezera kuphunzira kuchokera ku maganizo anu okhudzana ndi kupititsa patsogolo njira zopewera kutenga ndi kuchepetsa matenda m'zipatala ndi kunyumba.

Zokambiranazi zitenga pafupi-fupi ola imodzi ndi mphindi makumi atatu – kutengera ndi zimene mukuyenera kulankhula. Tilemba maganizo amene takambirana i ndipo, ngati mukuvomereza, zokambirana zijambulidwa ndicholinga chofuna kusunga uthenga umene mwalankhula molondola. Pa nthawi imene makina ojambulira mau ayatsidwa, getsi lofiira liyaka. Ngati mukufuna kunena china chilichonse 'chimene simukufuna kuti chisajumbulidwe'' palibe vuto, chonde adziwitseni otsogolera zokambirana kapena olemba zokambirana. Zojambula pa zokambiranazi zidzagwiritsidwa ntchito ndi gulu la anthu la kafukufuku lokha: Palibe munthu wina amene adzamva mau anu. Sitikulemba maina anu pano, ndipo palibe amene adzakuzindikirani kudzera mu malipoti ena aliwonse amene adzalembedwa mu kafukufukuyu. Zolembedwa zonse pa zokambiranazi zidzasungidwa motetezeka. Ndinu omasuka kuyankha moonjezera kapena mwachidule m'mene mungafunire, kudumpha mafunso ena amene simukufuna kuyankha, kapena kupumira kapena kusiya kuyankha mafunso nthawi ina iliyonse ngati kuli kofunikira kutero.

- *Tidzagawana nanu lipoti la chidule pa zopezeka zofunikira za kafukufuku wathu.*
- *Kodi pali wina aliyense amene ali ndi mafunso?*
- *Tiyeni tiyambe ndi kukhazikitsa malamulo.*
  - ✓ Malamulo okhazikitsidwa ndi gulu, monga
    - Munthu m'modzi yekha azilankhula nthawi imodzi.
    - Lankhulani momveka bwino
    - Ndikofunikira kuti timve malingaliro ndi maganizo a wina aliyense. Palibe yankho lolondola kapena lolakwika pa mafunso –ndi maganizo chabe, zokumana nazo ndi malingaliro, zonse zimene zili zofunikira.
    - Ndikofunikira kuti tonse timve mbali zonse za nkhani – zabwino ndi zoipa.
    - Chinsinsi ndichotsimikizika. "zokambirana mu chipinda zimatsala muchipinda momo."
    - THIMITSANI MAFONI A M'MANJA
  - ✓ Chiloezo
  - ✓ Pemphani anthu amu gulu kuti aliyense azifotokeze yekha pogwiritsa ntchito maina awo oyamba.

Uthenga wokhudzana ndi wotenga nawo mbali – chonde gwiritsani ntchito dzina loyamba potchulana muzokambirana

Kodi ndingatsimikize kuti ndinu okondwa kuti ndiyambe kujambula mchezowu? Chabwino. Zikomo

Zotsogolera zokambirana za pa gulu (FGD)

'Pakali pano ndifotokoza za mitu ina yokambirana ; umodzi pa nthawii; yokhudzana ndi kupewa kutenga matenda ndi kuchepetsa matenda, ndipo ndikukhulupilira kuti mukambirana mitu imeneyi pamodzi'

Udindo pa ntchito/maphunziro/machezedwe ndi ogwira nawo ntchito za umoyo

- Tiuzeni za udindo wanu pa ntchito imene mumagwira ku chipatala kuno kuphatikizapo zimene ntchito yanu imakhudzana nazo. Mumadziwa bwanji za zinthu zimene mukuyenera kuchita?
- Tifotokozereni za zimene zimachitika pa tsiku; pa nthawi imene mukugwira ntchito mu chipatala. Kodi ndi zipangizo ziti zimene zilipo/zimene palibe?
- Kodi ndi ndani amene amakuuzani za zimene mukuyenera kuchita? Ngati muli ndi mafunso okhudzana ndi ntchito yanu mukakhala ku chipatala, kodi mumamufunsa ndani?

Approved by College of Medicine 17-May-2023 (COMREC) Research and Ethics Committee

- Ndi zothandizira ntchito ziti/zothandizira ntchito zooneka ndi maso zimene muli nazo? Tiuzeni zokhudzana ndi maphunziro ena aliwonse amene mwakhala nawo kuti mukwanitse kugwira ntchito yanu ku chipatala. Kodi linali liti? Kodi ndimowirikiza bwanji?
- Tiuzeni za m'mene mumachezera ndi ogwira nawo ntchito za umoyo – Ogwira ntchito za umoyo/amene amayendetsa zochitika mu chipatala/odwala/munthu wina aliyense
- Tiuzeni zina mwa zovuta zimene mumakumana nazo pamene mukusamalira mu chipatala.

Zodziwa pa nkhani yopewa ndi kuchepetsa matenda

- Kodi mumamvetsa bwanji mau oti: kupewa kutenga/kuchepetsa/ kuchiza matenda?
- Kodi mukudziwa kuti anthu amatenga matenda akabwera ku chipatala?
- Kodi mukuganiza kuti anthu amatenga bwanji matenda? Kodi ndi njira ziti zimene tingapewe kuti anthu asatengere matenda? Ndindani amene mukuganizira kuti ali pa chiopsyezo chotenga matenda?
- Kodi mumadziwa bwanji kuti wina ali ndi matenda?

Maganizo pa m'mene nkhani zopewera ndi kuchepetsa matenda zingakonzedwere mwatsopano (IPC)

- Kodi ndi chani chimene chingakuthandizireni kugwira ntchito yanu bwino?
- Kodi ndi ndani amene angakuthandizireni?
- Kodi mungatengepo gawo lanji kuti mukonze mwatsopano nkhani zopewa kutenga ndi kufalitsa matenda (IPC)?

M'ndandanda wa mitu ya nkhani pa zimene zaonedwa mu zokambirana

Zimene zaonedwa mu zokambirana zidzatidziwitsa mafunso amene akuyenera kufunsidwa ndi wotsogolera zokambirana mwahitsanzo ., "Tinaona (izi zikuchitika) ku ma wodi ndipo tikufuna kudziwa ngati mungatiuze chifukwa chake …".

Mathero

Tikupita kumapeto kwa zokambirana zathu. Kodi pali china chake chimene wina wina angafune kuonjezera pa nkhani yopitisa patsogolo njira zopewera ndi kuchepetsa matenda?

- ✓ **Lakhulani mwachidule mfundo zikulu-zikulu zolakhulidwa ndi otenga nawo mbali**
- ✓ **Athokozeni otenga nawo mbali**

Approved by
College of Medicine
17-May-2023
(COMREC)
Research and Ethics Committee
