# Supplementary material for: Appraising the infection prevention and control practices at two referral hospitals in Malawi: a mixed methods situational analysis
Source: Antimicrob Resist Infect Control. 2026 Apr 6;15:76. doi: 10.1186/s13756-026-01742-7 (PMC13188669; doi:10.1186/s13756-026-01742-7)
Supplement: Supplementary file 5 — Supplementary Material 5 [file 13756_2026_1742_MOESM5_ESM.pdf]

Zoonjezera 6a ndi 6b: Zokambirana za pa gulu: Chikalata chotsogolera zokambirana: Woyang'anira wodwala

Chikalata chotsogolera zokambirana chikuyenera kusintha pakadutsa nthawi kutengera uthenga wina umene watoleredwa kuphatikizapo zimene zaonedwa. Choncho mitu yokambirana ikhoza kukhala yotsatirayi:)

Chikalata cha Zokambirana za pagulu	
**GAWO 1: UTHENGA WOKHUDZANA NDI WOTENGA NAWO MBALI**	
Lembani uthenga wokhudzana ndi wotenga nawo mbali wina aliyense.	
**GAWO 2: MAU OYAMBA A ZOKAMBIRANA ZA PA GULU**	
**ID ya Chipatala** [______]	**Zilembo zoyambilira za dzina la wotsogolera zokambirana** [___|____|____]
**Nambala ya ID ya zokambirana za pa gulu (FGD)** [____|_____|_____]	**Zilembo zoyambilira za dzina la wolemba zokambidwa pa mchezo (ngati alipo)** [___|____|____]
**Tsiku:** [___|___] / [___|____] / -[____|____] tsiku mwezi chaka	**Nthawi yoyambira** [____|____]: [____|_____] **Nthawi yomalizira** [____|____]: [____ |____]

Mau oyamba

Ine ndi ______________________________ ndi kuchokera ku ______________________ (Wotsogolera zokambirana)

Ine ndi______________________________ ndi kuchokera ku ______________________ (Wolemba zokambirana pa mchezo)

Zikomo kwambiri chifukwa chotenga nthawi yanu kulankhula ndi ine lero. Dzina langa ndi [ ] ndipo ndine m'modzi wa mugulu la kafukufuku wa IPC-Implement. Tisanayambe zokambiraa, ndikufuna nditsimkize ngati kmwalandira tsamba la chikalata cha uthenga ndi chikalata cha chilolezo?

Mongokumbutsa, kafukufukuyu cholinga chake ndikufufuza m'mene tingapititsire patsogolo njira zopewera kutenga ndi kuchepetsa matenda m'Malawi (IPC) ndi choliga chofuna kupititsa patsogolo chitetezo ndi chisamaliro chabwino kwa odwala. Takupemphani kuti mutenge nawo mbali mu zokambirana za tsiku la lero chifukwa tikuyembekezera kuphunzira kuchokera ku maganizo anu okhudzana ndi kupititsa patsogolo njira zopewera kutenga ndi kuchepetsa matenda m'zipatala ndi kunyumba.

Approved by College of Medicine 17-May-2023 (COMREC) Research and Ethics Committee

Zokambiranazi zitenga pafupi-fupi ola imodzi ndi mphindi makumi atatu– kutengera ndi zimene mukuyenera kulankhula. Tilemba maganizo amene takambirana i ndipo, ngati mukuvomereza, zokambirana zijambulidwa ndicholinga chofuna kusunga uthenga umene mwalankhula molondola. Pa nthawi imene makina ojambulira mau ayatsidwa, getsi lofiira liyaka. Ngati mukufuna kunena china chilichonse 'chimene simukufuna kuti chisajumbulidwe'' palibe vuto, chonde adziwitseni otsogolera zokambirana kapena olemba zokambirana. Zojambula pa zokambiranazi zidzagwiritsidwa ntchito ndi gulu la anthu la kafukufuku lokha: Palibe munthu wina amene adzamva mau anu. Sitikulemba maina anu pano, ndipo palibe amene adzakuzindikirani kudzera mu malipoti ena aliwonse amene adzalembedwa mu kafukufukuyu. Zolembedwa zonse pa zokambiranazi zidzasungidwa motetezeka. Ndinu omasuka kuyankha moonjezera kapena mwachidule m'mene mungafunire, kudumpha mafunso ena amene simukufuna kuyankha, kapena kupumira kapena kusiya kuyankha mafunso nthawi ina iliyonse ngati kuli kofunikira kutero.

- *Tidzagawana nanu lipoti la chidule pa zopezeka zofunikira za kafukufuku wathu.*
- *Kodi pali wina aliyense amene ali ndi mafunso?*
- *Tiyeni tiyambe ndi kukhazikitsa malamulo.*
  - ✓ Malamulo okhazikitsidwa ndi gulu, monga
    - Munthu m'modzi yekha azilankhula nthawi imodzi.
    - Lankhulani momveka bwino
    - Ndikofunikira kuti timve malingaliro ndi maganizo a wina aliyense. Palibe yankho lolondola kapena lolakwika pa mafunso –ndi maganizo chabe, zokumana nazo ndi malingaliro, zonse zimene zili zofunikira.
    - Ndikofunikira kuti tonse timve mbali zonse za nkhani – zabwino ndi zoipa.
    - Chinsinsi ndichotsimikizika. "zokambirana mu chipinda zimatsala muchipinda momo."
    - THIMITSANI MAFONI A M'MANJA
  - ✓ Chiloezo
  - ✓ Pemphani anthu amu gulu kuti aliyense azifotokeze yekha pogwiritsa ntchito maina awo oyamba.

Uthenga wokhudzana ndi wotenga nawo mbali – chonde gwiritsani ntchito dzina loyamba potchulana muzokambirana

Kodi ndingatsimikize kuti ndinu okondwa kuti ndiyambe kujambula mchezowu? Chabwino. Zikomo.

Zotsogolera zokambirana za pa gulu (FGD)

'Pakali pano ndifotokoza za mitu ina yokambirana ; umodzi pa nthawii; yokhudzana ndi kupewa kutenga matenda ndi kuchepetsa matenda, ndipo ndikukhulupilira kuti mukambirana mitu imeneyi pamodzi'

Zodziwa pa nkhani yopewa di kuchepetsa matenda

- Mungafotokoze bwanji chisamaliro chabwino?
- Mumavetsa bwanji zokhudzana ndi kupewa kutenga /kuchepetsa/kuchiza matenda?
- Kodi mukudziwa za anthu otenga matenda akabwera ku chipatala?
- Kodi mukuganiza kuti anthu amatenga bwanji matenda? Kodi ndi njira ziti zimene tingapewe kuti anthu asatenge matenda? Ndindani amene mukuganizira kuti ali pa chiopsyezo chotenga matenda?
- Kodi mumadziwa bwanji kuti wina ali ndi matenda? Kodi alipo amene angapereke zitsanzo za nthawi imene anadziwa kuti wina ali ndi matenda? Ndipo chinachitika ndi chani?

Zokumana nazo kuchipatala komanso kufuna chisamaliro

- Tiuzeni zimene munakumana nazo kuchipatala panthawiyo. Ndi malangizo/uthenga wanji umene munapatsidwa pa m'mene mungasamalire wodwala wanu? Kodi munalandira liti malangizowa?

Approved by College of Medicine
17-May-2023
(COMREC) Research and Ethics Committee

Anapereka ndi ndani?Kodi zinali zomveka? Kodi munakambirana izi ndi oyang'anira odwala ena? Ndi wodwala?<br>o Kodi ndi uthenga /malangizo ati amene mungafune kulandira pamene muli ku chipatala?<br>o Ndi chani chimene chinalipo chimene chinakuthandizirani ku chipatala zokuthandizirani kusamalira wodwala? Ndi chani chimene panalibe kapena chimene chingakonzedwe?<br>o Kodi ogwira nanu ntchito alankhula nanu bwanji?<br>**Chisamaliro cha odwala kunyumba**<br>o Kodi mudzayang'anira bwanji wodwala wanu mukabwelera kunyumba? Kodi mudzakumana ndi mavuto pofuna kuteteza wodwalayo kuti asatenge matenda? Ngati inde, tafotokozani.<br>o Kodi ndi uthenga wanji umene anthu amamva ku dera pa nkhani yokhudzana ndi kupewa matenda? Kodi mukuona kuti tikhoza kudziwitsa bwino bwanji anthu ku madera za m'mene angapewere kutenga ndi kuchhepetsa matenda? Kodi ndi ndani amene amafunika uthengawu?<br>**M'ndandanda wa mitu ya pa nkhani pa zimene zaonedwa mu zokambirana**<br>Zimene zaonedwa mu zokambirana zidzatidziwitsa mafunso amene akuyenera kufunsidwa ndi wotsogolera zokambirana mwahitsanzo ., "Tinaona (izi zikuchitika) ku ma wodi ndipo tikufuna kudziwa ngati mungatiuze chifukwa chake …".
**Mathero**<br>Tikupita kumapeto kwa zokambirana zathu. Kodi pali china chake chimene wina wina angafune kuonjezera pa nkhani yopitisa patsogolo njira zopewera ndi kuchepetsa matenda?
✓ **Lakhulani mwachidule mfundo zikulu-zikulu zolakhulidwa ndi otenga nawo mbali**<br>✓ **Athokozeni otenga nawo mbali**

Approved by
College of Medicine
17-May-2023
(COMREC)
Research and Ethics Committee
